# Comparative Analysis of Six Chloroplast Genomes in *Chenopodium* and Its Related Genera (*Amaranthaceae*): New Insights into Phylogenetic Relationships and the Development of Species-Specific Molecular Markers

**DOI:** 10.3390/genes14122183

**Published:** 2023-12-06

**Authors:** Zixiang Wei, Fangjun Chen, Hongxia Ding, Wenli Liu, Bo Yang, Jiahui Geng, Shihua Chen, Shanli Guo

**Affiliations:** 1 College of Life Sciences, Yantai University, Yantai 264005, China; weizx2021@s.ytu.edu.cn (Z.W.); fangjunchen202312@163.com (F.C.); ding202304@gmail.com (H.D.); wenli_liu1204@163.com (W.L.); yangbo9803@163.com (B.Y.); gengjiahui2022@163.com (J.G.); 2 College of Grassland Science, Qingdao Agricultural University, Qingdao 266109, China

**Keywords:** *Chenopodium*, *Atriplex*, chloroplast genome, phylogenetic relationship, divergence time, species identification, molecular marker

## Abstract

Species within the genus *Chenopodium* hold significant research interest due to their nutritional richness and salt tolerance. However, the morphological similarities among closely related species and a dearth of genomic resources have impeded their comprehensive study and utilization. In the present research, we conduct the sequencing and assembly of chloroplast (cp) genomes from six *Chenopodium* and related species, five of which were sequenced for the first time. These genomes ranged in length from 151,850 to 152,215 base pairs, showcased typical quadripartite structures, and encoded 85 protein-coding genes (PCGs), 1 pseudogene, 37 tRNA genes, and 8 rRNA genes. Compared with the previously published sequences of related species, these cp genomes are relatively conservative, but there are also some interspecific differences, such as inversion and IR region contraction. We discerned 929 simple sequence repeats (SSRs) and a series of highly variable regions across 16 related species, predominantly situated in the intergenic spacer (IGS) region and introns. The phylogenetic evaluations revealed that *Chenopodium* is more closely related to genera such as *Atriplex*, *Beta*, *Dysphania*, and *Oxybase* than to other members of the *Amaranthaceae* family. These lineages shared a common ancestor approximately 60.80 million years ago, after which they diverged into distinct genera. Based on InDels and SNPs between species, we designed 12 pairs of primers for species identification, and experiments confirmed that they could completely distinguish 10 related species.

## 1. Introduction

In the most recent APG IV (Angiosperm Phylogeny Group) plant classification system, the genus *Chenopodium* is categorized under the *Amaranthaceae* family, which encompasses approximately 250 species globally [1,2]. *Chenopodium* plants are mostly annual or perennial herbs, rarely semi-shrubs, with cystic hairs (powder) or cylindrical hairs, a small quantity of glandular hairs or completely hairless, and very low odor. They generally grow in arid or semi-arid areas and have tolerance to multiple stresses, including a strong salt tolerance. These characteristics make *Chenopodium* plants have extremely high scientific research value [3,4,5,6]. *Chenopodium* plants also have a high nutritional value. They can not only be grown as pseudocereals but also eaten as leafy vegetables [7]. Among them, the leaves of *C. album* are an important source of vitamins and trace elements in certain areas [8]. *C. quinoa*, known as a “superfood”, is not only rich in fatty acids, vitamins, minerals, dietary fiber, and proteins, containing many amino acids, but also rich in carotenoids, vitamin C, and phenolic compounds. These compounds contribute to the prevention of conditions such as allergies and cardiovascular diseases [9,10,11,12,13]. In some arid areas, they are also used as medicinal plants, fodder, and dye raw materials [14].

In addition, some plants of the related genera of *Chenopodium* also have various utilization values. For example, some plants from *Atriplex* can revegetate arsenic-contaminated saline–alkali soils, while effectively accumulating and preventing the spread of arsenic and reducing the risk of human exposure to arsenic [15]. Some compounds extracted from *Atriplex* can protect the chromosome damage caused by gamma radiation and can be used to develop new radioprotective drugs [16]. *D. ambrosioides* exhibits high antimicrobial and antioxidant activities and can enhance the antibacterial activity with the synergistic effect of antibiotics. At the same time, some compounds contained in it can achieve the purpose of killing mosquitoes by destroying the nervous system of insects and inhibiting the activity of key enzymes [17,18,19].

However, due to the high similarity and polymorphism of leaf shape, floral structure, and seed morphology and the existence of possible hybrid species, the classification and identification of *Chenopodium* and its related genera have become extremely complex and controversial, which severely hinders the development of related scientific research and the efficient utilization of *Chenopodium* plants [20,21,22,23].

Previous investigations in *Chenopodium* primarily relied on conventional molecular markers for phylogenetic analysis and species identification. These markers include nuclear DNA amplification fragment length polymorphism, ribosomal DNA (rDNA) internally transcribed spacer sequence (ITS) variation, and chloroplast DNA (cpDNA) fragments [24,25,26,27,28]. However, these research methods have limitations due to the restricted information they provide regarding mutation sites. Consequently, these approaches may not fully address the requirements of related research [29].

Several scholars have suggested employing the complete cp genome as a prospective solution to address this issue [30,31,32,33]. The cp genome of closely related species is highly conserved in structure, yet it exhibits subtle differences. Furthermore, the longer sequences provide a wealth of mutation site information, thereby enhancing its capability for species identification [34,35,36,37]. Simultaneously, the cp genome of angiosperms is mostly maternally inherited, reducing gene pollution and ensuring the accurate transmission and storage of genetic information [38,39]. Some researchers have used the cp genome to mine mutation sites to distinguish closely related species (especially some weed relatives), or have used the cp genome to elaborate new perspectives on plant phylogenetic relationships and taxonomy. The importance and efficiency of chloroplast genomes in related research have been confirmed by more and more studies [40,41].

In this study, we obtain the complete cp genomes of six closely related species of *Chenopodium* and describe their basic characteristics in detail. By comparing with the sequence information of other species that have been published, we studied their phylogenetic relationship and divergence time, which provided valuable clues to understand the evolutionary history of the genus *Chenopodium*. In addition, we also developed a set of highly efficient molecular marker primers that can be used to accurately identify these closely related species; these molecular markers could be widely applied to assist in screening and identifying *Chenopodium* plants with specific genomic features, thereby accelerating the breeding process for new varieties of crops, such as *C. quinoa*.

## 2. Materials and Methods

### 2.1. Plant Material and DNA Isolation

We collected the young leaves of 6 plant species (*A. patens*, *A. prostrata*, *C. album*, *C. berlandieri*, *C. bryoniifolium*, and *L. franchetii*) from Yantai, Shandong Province, China (121°44′ E, 37°51’ N, 264,003). The identification of samples was based on the classification and external morphological diagnosis of the samples according to the Chinese Virtual Herbarium (CVH) and Flora of China [42]. The specimen voucher materials were preserved in the College of Life Sciences, Yantai University. The total DNA was obtained from plants through the modified CTAB method [43]. The quality of DNA was assessed using a NanoDrop spectrophotometer 2000 (Thermo Scientific, Waltham, MA, USA), and DNA integrity was verified through 1% gel electrophoresis.

### 2.2. DNA Sequencing, Assembly, and Annotation

To create libraries, 350 bp fragments were generated using paired-end sequencing on the Illumina HiSeq 2500 platform (Illumina Inc., San Diego, CA, USA).

To obtain clean data, Fastp 0.23.1 was used to remove ambiguous bases or low-quality bases from raw sequencing reads [44]. GetOrganelle v1.7.1 was utilized for the assembly of the cp genome, with the k-mer length parameter configured as follows: −k 21, 45, 65, 85, and 105 [45]. Bandage v0.81 and Gepard v1.40 were used to examine the cp gene composition loop and collinearity, respectively [46,47].

The cp genome was annotated by employing CPGAVAS2 and GeSeq. Subsequently, Apollo was used for proofreading [48,49,50]. To locate and annotate tRNA genes, tRNAscan-SE v2.0.7 was employed for analysis, and CPgview was employed to check the annotation of the rps12 gene [51,52]. Finally, the complete cp genome map was generated through Chloroplot [53]. The annotated cp genome sequence was stored in GenBank with the accession number OR374019-OR374024.

### 2.3. Genome Structure and Comparison Analysis

Combined with the published sequence information in the NCBI database, a sum of 16 complete cp genomes of *Chenopodium* and related species were used for comparative analysis. The analysis of SSRs was conducted using MISA v2.1 [54]. The parameters were set as follows: mononucleotides ≥ 10; dinucleotides ≥ 5; trinucleotides ≥ 4; and tetranucleotides, pentanucleotides, and hexanucleotides ≥ 3. REPuter was used to detect the four types of interspersed repeats, including forward repeats, palindromic repeats, reverse repeats, and complementary repeats [55]. The minimum repeat fragment size was set to 30 bp, and the Hamming distance was set to 3. The relative synonymous codon usage (RSCU) was calculated by CodonW v1.4.2, and the results were subsequently visualized by Tbtools v1.113 [56,57].

The Perl script CPJSdraw v1.0 was employed to compare and visualize the inverted repeat (IR) region boundary characteristics between the cp genomes of the 16 species of *Chenopodium* and its related genera [58]. Using *A. centralasiatica* as the reference genome, in the Shuffle-LAGAN model of mVISTA, the cp genomes were compared to understand the sequence variations among closely related species [59,60]. DnaSP v5.10 was employed to analyze the nucleotide diversity of 111 gene regions and 135 intergenic regions (including introns) shared by all closely related species to obtain high information content sites [61].

### 2.4. Phylogenomic Analysis and Divergence Time Estimation

For the purpose of acquiring an in-depth understanding of the phylogenetic relationship among the species of *Chenopodium* and its related genera, data were downloaded from other species under the *Amaranthaceae* from the NCBI database as a supplement. In total, 155 complete cp genome sequences from 69 species in 24 genera were obtained, and their sequence starting points and small single copy (SSC) region directions were corrected and unified. Among them, five species of *Plumbaginaceae* were employed as outgroups. The alignment of all cp genome sequences was performed using MAFFT v7.50 and then trimmed using trimAl v1.41; the phylogenetic relationships were inferred based on the maximum likelihood (ML) and Bayesian inference (BI) [62,63]. The best nucleic acid replacement model was selected using Model Finder, and the ML tree was reconstructed using IQ-TREE 2.03 under the TVM + F + R3 model [64,65]. The ML branch support was evaluated by 1000 ultrafast guided repetitions.

The BI tree was reconstructed using MrBayes 3.2.7 [66]. A total of 20 million generations were iterated with the GTR + F + I + G4 model, with one cold chain and three heated chains, and the initial 25% of the sampled data was discarded as burn-in. Finally, the average standard deviation of split frequencies (ASDSF) < 0.01, the effective sample size was >200, and the potential scale reduction factor (PSRF) was close to 1, reaching convergence.

Based on the ML tree, the divergence time of the 16 *Chenopodium* related species (each species retains only one sequence) in Clade I was calculated using the Markov chain Monte Carlo (MCMC) tree program in PAML [67]. According to previous studies, five fossil calibration points were used to limit each node, of which 81.3–97.0 Ma was used as the age of the root node [68]. The independent rates that follow the lognormal distribution were selected as the clock model. We selected HKY85 as the nucleic acid replacement model, in which the alpha for gamma rates at the sites was set to 0.5. The birth–death process was used to generate uniform node age priors in the tree, using the default parameters (l = 1, m = 1, and s = 0.1). We ran the Markov chain Monte Carlo program for 200 million generations, sampling every 100 cycles. The first 25% of the samples was discarded as burn-in. Finally, Tracer v1.72 was used to check the output file to confirm the convergence based on ESS > 200.

### 2.5. Development and Validation of InDel and dCAPS Markers

Based on the variable regions in the cp genomes of *Chenopodium* and its related species, a series of molecular markers were developed to distinguish a total of 10 related species. Primer3 4.1.0 and dCAPS Finder 2.0 were used to design primers for the amplification of InDel markers and SNP-based dCAPS markers in the conserved regions near the variant sites [69,70].

Polymerase chain reaction (PCR) was performed in a total volume of 10 μg. It contained 5 μg 2 × Taq Master Mix (Vazyme, Nanjing, China), 1 μg DNA template, 0.5 μg of upstream and downstream primers, and 3 μg of ddH2O. The PCR was executed with a T100TM thermal cycler (Bio-Rad, Hercules, CA, USA). The settings were established as: pre-denaturation at 95 °C for 5 min, followed by 35 cycles of denaturation at 95 °C for 15 s, annealing at 55–58 °C for 15 s, extension at 72 °C for 1–3 min, and finally extension at 72 °C for 7 min. The PCR products were electrophoresed in 2–3% agarose gel, and the PCR products of the dCAPS marker were first reacted with the restriction enzyme (BsrI) at 65 °C for 1 h. Finally, Gel DocTM XR + gel imager (Bio-Rad, Hercules, CA, USA) was used for visualization.

## 3. Results

### 3.1. Genome Structure

**Figure 1:**
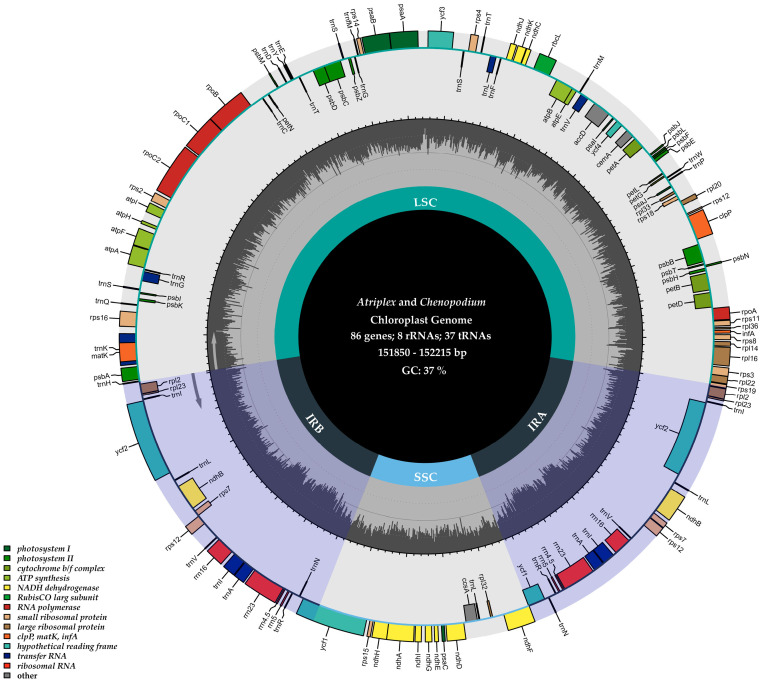
Complete cp genome map of *Atriplex* and *Chenopodium*. Genes with different functions were divided by color. Genes inside and outside the circle were transcribed clockwise and counterclockwise, respectively.

We sequenced and assembled the cp genomes of six *Chenopodium* species and related genera, and then compared these to the existing cp genomes of the associated genera in the NCBI database. The lengths of these newly sequenced cp genomes varied from 151,850 bp (*A. patens*) to 152,215 bp (*A. prostrata*). Both *Chenopodium* and *Atriplex* cp genomes presented a typical quadripartite structure (Figure 1). The IR region (varying from 25,161 bp (*A. patens*) to 25,192 bp (*C. bryoniifolium*)) were separated by a large single copy (LSC) region (ranging from 83,675 bp (*C. bryoniifolium*) to 83,887 bp (*A. prostrata*)) and an SSC region (ranging from 17,811 bp (*A. patens*) to 18,131 bp (*C. berlandieri*)). The overall GC content in the cp genomes of *Chenopodium* and *Atriplex* was found to range between 37.25% and 37.3%. Nevertheless, there was a non-uniform distribution of the GC content in distinct genomic regions. Precisely, the GC content within the IR region (42.72–42.81%) exceeded that of both the LSC (35.30–35.40%) and the SSC regions (30.82–31.02%), which is consistent with other *Chenopodium* and related species previously reported [71,72]. Several studies proposed that the elevated GC content observed in the IR region possibly stems from the higher prevalence of GC-rich genes, such as rRNA and tRNA, within these genomic sections [73,74,75].

The cp genomes of *Chenopodium* and *Atriplex* encode 131 genes (totaling 113 unique genes), including 85 protein-coding genes (PCGs), 1 pseudogene (a shorter incomplete copy of ycf1), 37 transfer RNA (tRNA) genes, and 8 ribosomal RNA (rRNA) genes. The order and orientation of these genes were the same in all species, which is in accordance with the outcomes documented in the related genera, such as *Beta*, *Dysphania*, and *Oxybasis* (Table 1). Among the PCGs, 48 genes were associated with photosynthesis, while 75 genes were involved in transcription and translation processes (Table 2). In total, 17 genes contained one intron (*rps16*, *atpF*, *rpoC1*, *petB*, *petD*, *rpl16*, *ndhB* (×2), *ndhA*, *trnK-UUU*, *trnG-UCC*, *trnL-UAA*, *trnI-GAU* (×2), *trnA-UGC* (×2), and *trnV-UAC*) and 3 genes contained two introns (*ycf3*, *clpP*, and *rps12*). Additionally, *rps12* underwent trans-splicing, involving the concatenation of two exons. The 5′ exon is situated within the LSC region, while the 3′ exon is positioned in the IR region. A total of 17 genes, including 6 PCGs (*rpl2*, *rpl23*, *ycf2*, *ndhB*, *rps7*, and *rps12*), 7 tRNA genes (*trnI*, *trnL*, *trnV*, *trnI*, *trnA*, *trnR*, and *trnN*), and all 4 rRNA genes (*rrn16*, *rrn23*, *rrn4.5*, and *rrn5*), were found to be duplicated in the inverted repeat (IR) region.

### 3.2. Repeat Sequence Analysis

**Figure 2:**
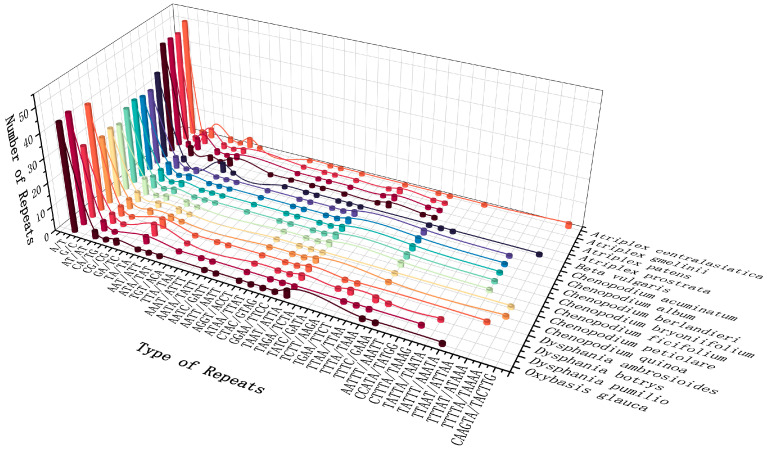
Types and numbers of simple sequence repeats in the cp genomes of 16 *Chenopodium* and related species.

SSRs with 1–6 bp repeat nucleotide units are widely distributed in plant cp genomes [76]. The number of SSRs in the 16 species of *Chenopodium* and related genera ranged from 44 (*C. quinoa* and *C. petiolare*) to 74 (*A. centralasiatica*). Except for pentanucleotide SSRs, which were not found in *A. prostrata*, *A. patens*, and *A. gmelinii*, all species contain mononucleotide, dinucleotide, trinucleotide, tetranucleotide, and pentanucleotide SSRs. However, hexanucleotide SSRs only exist in *A. centralasiatica*. Among all SSRs, A/T and AT/AT were the most abundant, accounting for 65.30% and 7.53% respectively. Research has demonstrated that a high AT content in the cp genome of higher plants is a prevalent phenomenon [77,78,79]. SSRs were predominantly located within the intergenic spacer (IGS) of the LSC and SSC regions, with a smaller fraction being distributed within the coding regions of *ycf1*, *ycf2*, and *rrn23* in the IR region (Figure 2).

**Figure 3:**
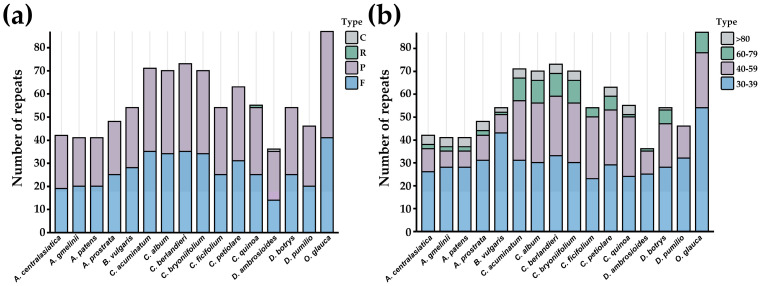
Interspersed repeats in the cp genomes of 16 *Chenopodium* and related species. (**a**) Number of 4 types of repeats. (**b**) Number of different repetition repeats.

Within the cp genomes of 16 species, the count of interspersed repeats varied between 36 (*D.ambrosioides*) and 87 (*O.glauca*). Forward and palindrome repeats were identified across all species, whereas reverse and complementary repeats were exclusively observed in *C. quinoa* and *D. ambrosioides*, respectively. The majority of the interspersed repeats were forward repeats (*n* = 14–41) and palindrome repeats (*n* = 21–46), accounting for 47.62% and 52.15%, respectively, and the size was mainly concentrated in 30–39 bp (Figure 3).

### 3.3. Analysis of Codon Usage Bias

**Figure 4:**
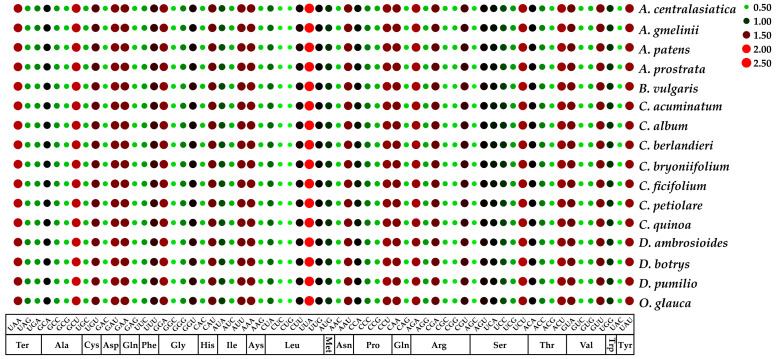
RSCU values of the chloroplast genomes of the 16 *Chenopodium* and related species. Red and green represent higher and lower RSCU values, respectively.

The gene coding sequences in the 16 closely related *Chenopodium* species consist of 64 codons, with 61 encoding 20 amino acids, while the remaining 3 (UAA, UAG, and UGA) serve as stop codons. Leucine exhibits the highest frequency, while cysteine demonstrates the lowest frequency among all amino acids. The RSCU indicates the codon usage frequency relative to the expected frequency [80]. In this study, 30 codons exhibited RSCU values exceeding 1, indicating values higher than expected, whereas 32 codons showed RSCU values below 1, signifying values lower than expected. Furthermore, the RSCU values of the remaining two codons, AUG and UGG, equaled 1. In addition, the bases of the high-frequency codons in the third position showed a strong A/T preference (Figure 4).

### 3.4. Genome Comparison and Nucleotide Diversity

**Figure 5:**
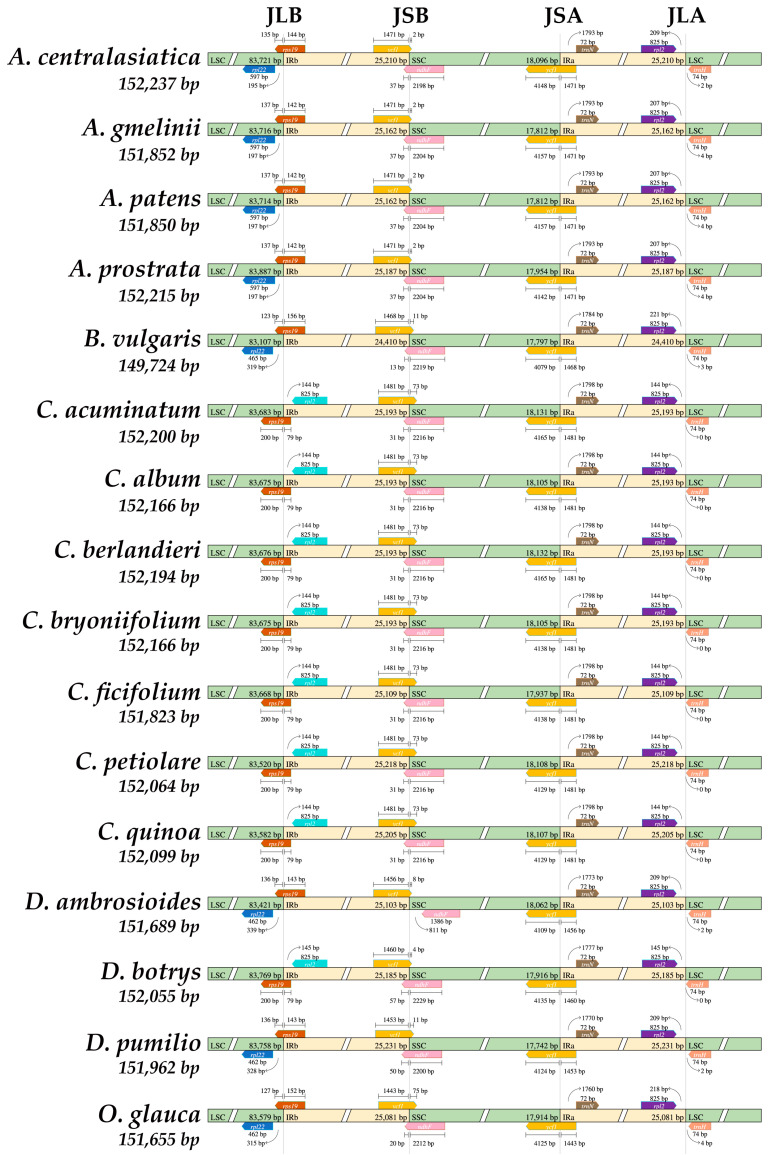
Comparison of the boundaries of the LSC, SSC, and IR regions among the 16 *Chenopodium* and related species.

In the field of plant evolution research, the dynamic alteration of the IR region within the cp genome stands as a pivotal event. It holds the potential to induce variations in both the size and genetic composition of the cp genome [81]. The comparative analysis of seven species of *Chenopodium* and nine species of related genera showed that, except for the obvious contraction of the IR region of *Beta*, the cp genome structure and gene sequence were substantially conserved within and among the genera, except for a subtle variation in the IR/SC region junctions. The LSC/IRb (JLB) junctions of all 16 species are located within *rps19*, but the part of *rps19* located in the IRb region is less in *Chenopodium* (79 bp), while all other closely related species, except for *D. botrys* (79 bp), are more (142–156 bp). The IRb/SSC (JSB) junctions are located within *ndhF* and range from 2198 to 2229 bp, except for *D. ambrosioides* (811 bp from *ndhF*). The SSC/IRa (JSA) junction is located at 1443–1481 bp inside *ycf1*. The IRa/LSC (JLA) junction is located between *rpl2* and *trnH* and is 0–4 bp away from *trnH* (Figure 5).

**Figure 6:**
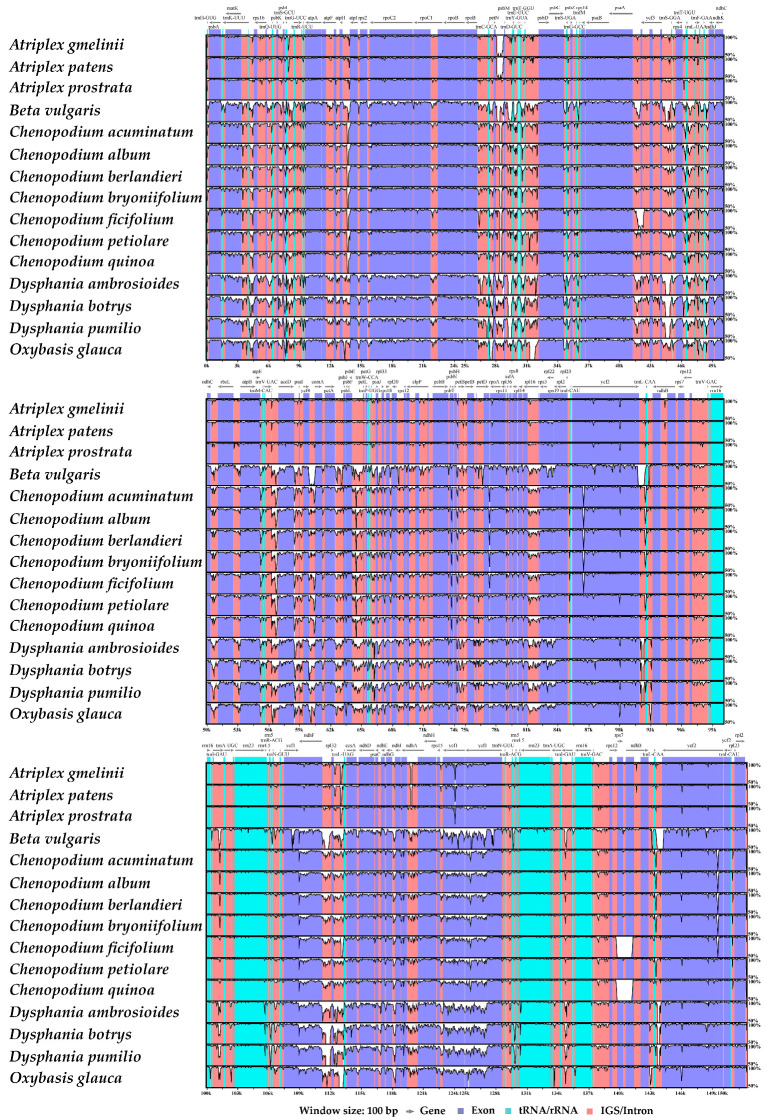
Based on the reference cp genome of *A.centralasiatica*, the cp genomes of the 16 *Chenopodium* and related species were compared using mVISTA. The *y*-axis represents the percent identity within 50–100%. Genome regions were color-coded as exon (purple), Trna/rRNA (blue), and intergenic spacer region and intron (pink). Gray arrows display the gene orientation.

**Figure 7:**
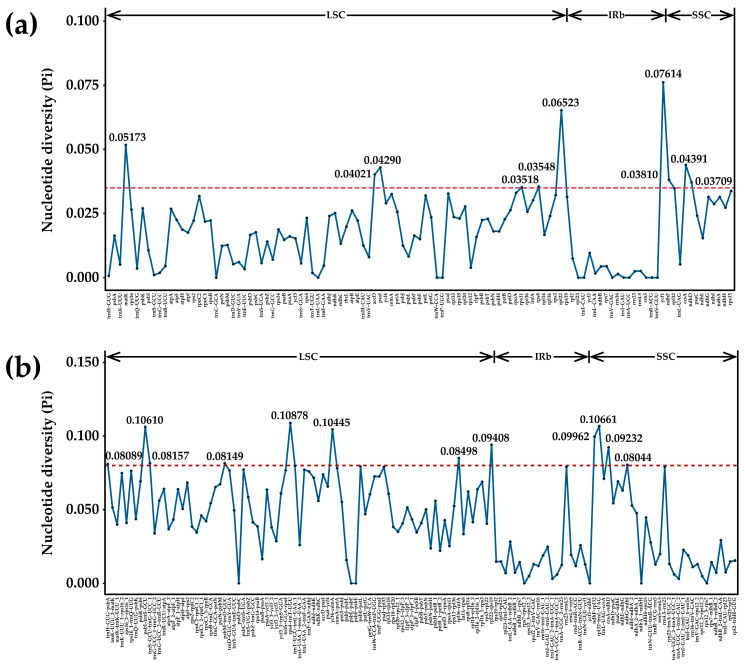
Comparison of the nucleotide diversity (Pi) of the cp genomes of the 16 *Chenopodium* and related species. (**a**) and (**b**) represent the gene region and intergenic spacer region (IGS), respectively, with 0.035 and 0.08 as the screening thresholds for the gene region and IGS, respectively (Table S1).

Taking *A. centralasiatica* as a reference, the cp genomes of the 16 species of *Chenopodium* and related genera were compared and analyzed (Figure 6). The cp genome is extremely conserved within the same plant genus, yet intergeneric differences persist. On the whole, the variation level of IGS and intron regions is higher than that of exon regions. A nucleotide diversity (Pi) analysis was further performed on 42 cp genomes of the above 16 species (Figure 7). The results show that the IGS region (average Pi = 0.04430) had higher polymorphism than the gene region (average Pi = 0.01810). The most variable gene regions included: *matK*, *accD*, *psaI*, *rps11*, *rps8*, *rpl22*, *ycf1*, *ndhF*, *ccsA*, and *ndhD* (PI > 0.035), six of which were situated in the LSC region, while four were positioned in the SSC region. The IGS regions with a large variation include: *trnH-GUG-psbA*, *psbI-trnS-GCU*, *trnS-GCU-trnG-UCC*, *psbM-trnD-GUC*, *rps4-trnT-UGU*, *ycf4-cemA*, *rpl36-infA*, *rpl22-rps19*, *ndhF-rpl32*, *rpl32-trnL-UAG*, *ccsA-ndhD*, and *ndhG-ndhI* (Pi > 0.080). Among them, eight were situated in the LSC region and four in the SSC region.

### 3.5. Phylogenomic Analysis and Divergence Time Estimation

**Figure 8:**
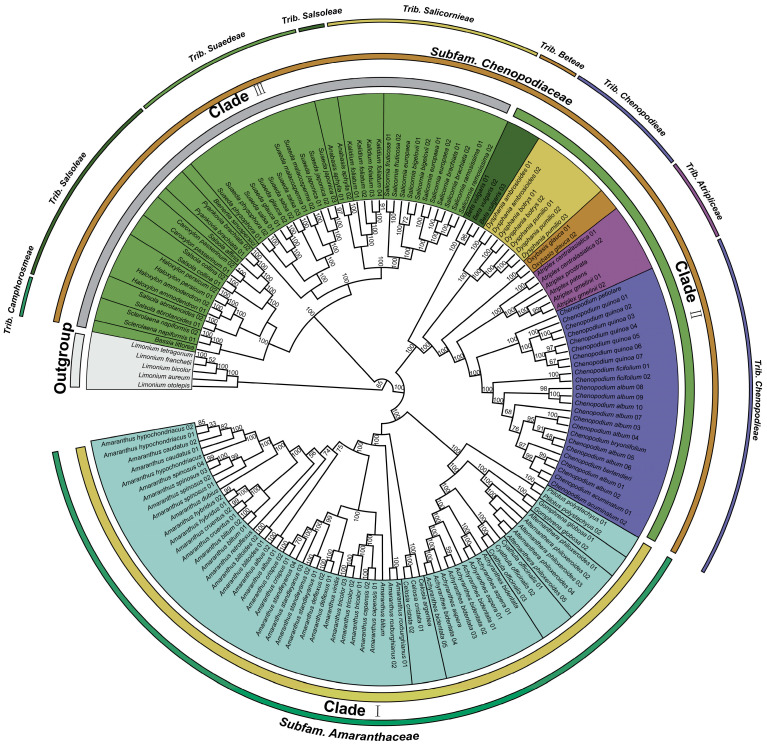
Maximum likelihood phylogenetic tree based on the complete cp genome. Several species of *Limonium* were outgrouped.

The cp genome plays a pivotal role in revealing the evolutionary relationships among plants [82]. Taking several species of *Limonium* as the outgroup, we reconstructed the phylogenetic trees of 63 species of *Amaranthaceae*, including *Chenopodium* (Table S2). The maximum likelihood (ML) trees and Bayesian inference (BI) trees had almost the same topological structure, with a strong bootstrap support (BS) and posterior probabilities (Figure 8). In the ingroup, all species were classified into three main clades (clade I, clade II, and clade III). Based on morphological studies (Flora of China), all species in clade I belong to *Subfam*. *Amaranthaceae*, while all species in clade II and clade III belong to *Subfam. Chenopodiaceae*. Among them, all the species in clade II are distributed in three tribes (*Trib. Atripliceae*, *Trib. Beteae*, and *Trib. Chenopodieae*), while the species in clade III are distributed in four other tribes (*Trib. Camphorosmeae*, *Trib. Salicornieae*, *Trib. Salsoleae*, and *Trib. Suaedeae*). In addition, all the species of *Chenopodium* and its four related genera (*Atriplex*, *Beta*, *Dysphania*, and *Oxybasis*) in clade II were clustered together as genera, forming five monophyletic groups. This result further supports the inference of other studies that *Chenopodium* is a monophyletic group [7].

**Figure 9:**
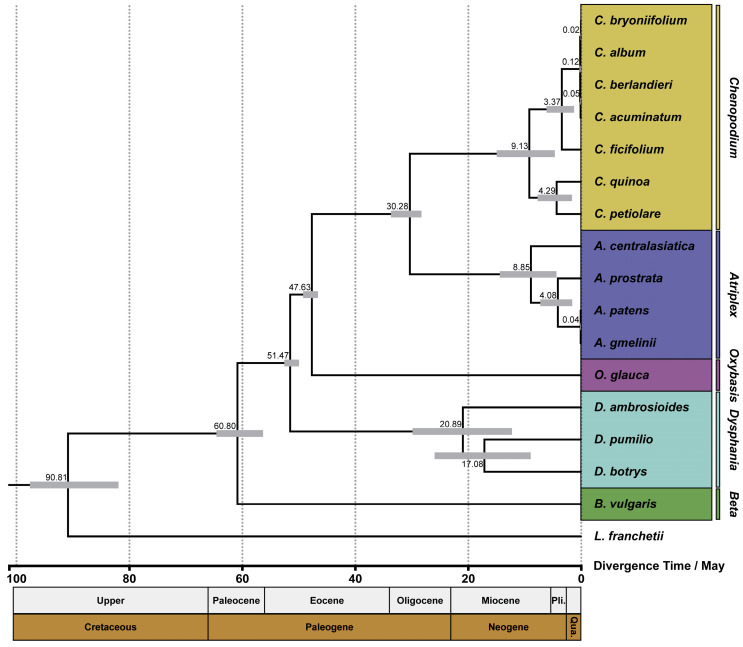
Estimation of the divergence time of the 16 related species. The gray bar and the number above it represent the 95% highest posterior densities and divergence time, respectively.

Based on the ML tree, we estimated the divergence time of the 16 species in clade I (Figure 9). The outcomes demonstrate that the 16 species of *Chenopodium* and related genera had a common ancestor in the Paleocene period at 60.80 Mya (95% highest posterior densities (HPD): 56.27–64.52 Mya). At the same time, *Beta* also split from other genera. After that, *Dysphania* and *Oxybasis* split from other species at 51.47 Mya (95% HPD: 49.91–52.51 Mya) and 47.63 Mya (95% HPD: 46.54–49.17 Mya), respectively. *Atriplex* was the closest relative to *Chenopodium* in all related genera; they were sister clades and split at 30.28 Mya (95% HPD: 28.22–33.62 Mya). The seven species of *Chenopodium* began to split in the Miocene period at about 9.13 Mya (95% HPD: 4.61–14.89 Mya) and separated into two branches represented by *C. quinoa* and *C. album. C. album* and *C. bryoniifolium* are two species that were extremely difficult to distinguish in morphological taxonomy. Their split occurred 20,000 years ago (95% HPD: 0–0.06 Mya) and is also the most recent.

### 3.6. Development and Validation of the InDel and dCAPS Markers

**Figure 10:**
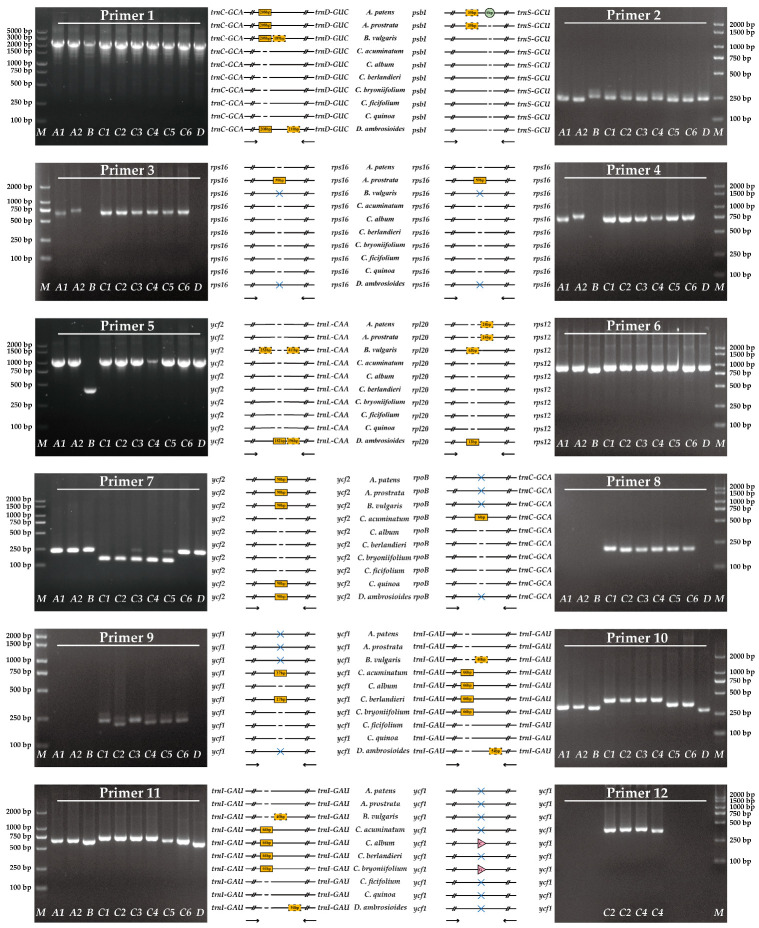
Verification of the primers based on the InDel and dCAPS markers. The lane M represents the 2000/5000 bp marker; A1–A2 are *A. patens* and *A. prostrata*, respectively; C1–C6 are *C. acuminatum*, *C. album*, *C. berlandieri*, *C. bryoniifolium*, *C. ficifolium*, and *C. quinoa*, respectively; and B and D are *B. vulgaris* and *D. ambrosioides*, respectively. The rectangles composed of solid lines and dotted lines represent the insertion and deletion of InDel, respectively; the circles and triangles represent SSRs and SNPs, respectively; the horizontal solid line and dotted line represent the conserved and variant sequences, respectively; the arrow indicates the direction of the primer; and the blue fork indicates that the primers are not applicable.

Based on the interspecific analysis, we designed a series of primers on the conserved region near the variant sites to amplify fragments with genetic marker information (Table S3). The results show that 12 pairs of primers could successfully amplify the expected polymorphic sequences and could completely distinguish 10 species of *Chenopodium* and related genera (Figure 10). Most of the primers were designed based on InDel markers. Among them, primers 1 and 2 can be used to distinguish *Atriplex* from other genus species, and primers 3 and 4 can further distinguish *A. patens* and *A. prostrata*. Primer 5 and primer 6 can be used to distinguish *Beta* species. Primer 7 can distinguish *Chenopodium* species, except for distinguishing *C. quinoa* from other species. Primer 8 can be used to distinguish *C. acuminatum* from other *Chenopodium* species, and primer 8 and primer 9 can further distinguish *C. berlandieri*. In addition to directly distinguishing *D. ambrosioides*, primers 10 and 11 can also distinguish *C. ficifolium* from other *Chenopodium* species. At the same time, the combination of primers 7 can further distinguish *C. quinoa*. Primer 12 is the only pair of primers based on dCAPs markers, which are more cumbersome than InDel markers. Because there are only two SNPs in the cp genome of *C. album* and *C. bryoniifolium*, we assembled and annotated their ITS sequences, but there was no difference after comparison.

## 4. Discussion

After sequencing and assembling, we obtained the complete cp genomes of six closely related *Chenopodium* species, five of which are published in this paper for the first time, which greatly enriches the genome database of *Chenopodium* species and facilitates their classification, evolutionary relationship inference, and species identification.

**Figure 11:**
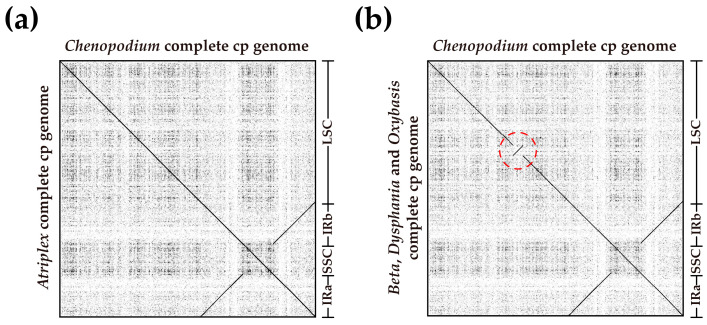
Dot plot of the inversions in the cp genomes of *Beta, Dysphania*, *and Oxybasis*. (**a**,**b**) represent the collinearity between *Chenopodium* and *Atriplex*, and between *Chenopodium* and *Beta*, *Dysphania*, *Oxybasis*, respectively. The interior of the red circle is the area where inversions occur.

Similar to previous studies, their lengths ranged from 151,850 to 152,215 bp and exhibited typical quadripartite structures [83,84]. The cp genomes of most terrestrial plants exhibit a nearly collinear sequence and genetic information, but there are also other occurrences, such as sequence inversion, gene loss, and IR region boundary contraction or expansion [85,86,87]. In the comparison of the cp genome of *Chenopodium* with other related genera, we found that there was an inversion of almost 6 kb in the LSC region of *Beta*, *Dysphania*, and *Oxybasis* (Figure 11). Studies have shown that this phenomenon is caused by tRNA activity or a high G + C content [88,89]. There was a *trnV* gene on the flanking of the region where the inversion occurs, but no significant change in the GC content was found.

The primary drivers behind cp genome size alterations are the contraction or expansion of the IR boundary [90,91,92]. The IR boundaries of *Chenopodium* and its related species are relatively conserved on most species, except for the obvious contraction on *Beta*, which also leads to the cp genome of *B. vulgaris*, which is about 2000 bp smaller than that of the other related species.

Repeat sequences exert a pivotal influence on the cp genome rearrangement and stabilization and population genetic diversity [93,94]. In this study, interspersed repeats and SSRs were widely distributed in all related species, and the proportion of repeat sequences in the IGS region was significantly greater than that in the CDS region, which also confirms the previous research results.

Previous studies on the phylogeny of *Chenopodium* based on cpDNA fragments (*trnL-trnF*, *matK-trnK*, *atpB*, *atpB-rbcL*, and *rbcL*) and ITS in rDNA presented some taxa conclusions that are different to our results, and the relationship was relatively vague [2,95,96]. Consequently, we reconstructed the phylogenetic tree utilizing the complete cp genome sequence. The results show that *Chenopodium*, *Atriplex*, *Beta*, *Dysphania*, and *Oxybasis* were clustered into a clade, and each of them became a monophyletic group. This also confirmed that the genetic distance between *Chenopodium* and *Beta* was closer than that of *Suaeda* and *Haloxylon*. In addition, the traditional plant classification method based on morphological characteristics included *Chenopodium* in the same tribe as *Dysphania* and *Oxybasis*, while *Atriplex* was classified as *Trib. Atripliceae*, which is obviously contrary to the phylogenetic outcomes derived from the complete cp genome, because *Chenopodium* and *Atriplex* have a closer genetic distance.

We used *Limonium franchetii*, which is also *Caryophyllales*, as the outgroup to estimate the evolutionary time of *Chenopodium* and its related species. The findings indicate that the divergence time between *Amaranthaceae* and the other families of *Caryophyllaceae* was 90.81 Mya, and the divergence time between *Chenopodium* and other genera was 30.28 Mya, which was different from the results of previous studies.

Previously, *rbcL*, *trnH-psbA*, and *matK* were regarded as critical barcodes for plant identification, but their resolution at the species level was inadequate [97]. The complete cp genome has more variable regions and has a higher resolution when used as a DNA barcode. Dependent on the complete cp genome, we developed InDel and dCAPS that can completely distinguish the 10 related species of *Chenopodium*. At the same time, a series of primers were designed based on these markers and verified by PCR experiments. These primers have a good repeatability and can be efficiently used for species identification and germplasm evaluation among the related species of *Chenopodium*. The ITS sequences of *Chenopodium* species were also assembled and we attempted to utilize these sequences for molecular marker development, but unfortunately, there are less variable sequences than the complete cp genome. For example, the ITS sequences of *C. album* and *C. bryoniifolium* are exactly the same and cannot be used for species discrimination.

In conclusion, this study introduced a significantly more efficient means for the identification of species within the *Chenopodium* genus and their closely related species.

## 5. Conclusions

In this investigation, the complete cp genomes of six species were assembled and annotated, and compared with the published sequences of related species. The outcomes reveal that the cp genomes of *Chenopodium* and its related species were highly conserved in structure and gene content, but there were also subtle variances. We identified a series of SSRs and highly variable regions from all *Chenopodium* and related species, and found that most of them were distributed in IGS regions and introns. The phylogenetic analysis showed that *Chenopodium* had the closest relationship with *Atriplex*, *Beta*, *Dysphania*, and *Oxybasis* and belonged to a monophyletic group compared with other *Amaranthaceae* species. They had a common ancestor 60.80 Mya, and since then, it gradually divided into different genera. Based on the interspecific variation sites, we designed 12 pairs of primers for species identification, and the experiments confirmed that they could completely distinguish 10 related species.

## Figures and Tables

**Table 1:** Summary of the cp genomes for the 16 *Chenopodium* and related species.

Accession	Species	Total	LSC	IR	SSC	Total	LSC	IR	SSC	Total	Protein-Coding	rRNA	tRNA
Number		Length (bp)	(bp)	(bp)	(bp)	GC Content (%)	GC Content (%)	GC Content (%)	GC Content (%)	Genes	Genes	Genes	Genes
NC_045304	*A. centralasiatica*	152,237	83,721	25,209	18,095	37.27	35.38	42.72	30.85	131	86	8	37
NC_059062	*A. gmelinii*	151,852	83,716	25,161	17,811	37.29	35.35	42.81	30.86	131	86	8	37
OR374023	*A. patens* *	151,850	83,714	25,161	17,811	37.29	35.35	42.81	30.86	131	86	8	37
OR374024	*A. prostrata* *	152,215	83,887	25,186	17,953	37.3	35.4	42.77	30.82	131	86	8	37
ON641300	*B. vulgaris*	149,724	83,107	24,409	17,796	37	34.97	42.84	30.44	131	86	8	37
NC_054154	*C. acuminatum*	152,200	83,683	25,192	18,130	37.25	35.3	42.72	31.01	131	86	8	37
OR374020	C. album *	152,166	83,675	25,192	18,104	37.25	35.3	42.72	30.99	131	86	8	37
OR374022	C. berlandieri *	152,194	83,676	25,192	18,131	37.25	35.3	42.72	31.02	131	86	8	37
OR374021	C. bryoniifolium *	152,166	83,675	25,192	18,104	37.25	35.3	42.72	30.99	131	86	8	37
NC_041200	*C. ficifolium*	151,823	83,668	25,108	17,936	37.26	35.31	42.75	31	131	86	8	37
OQ957163	*C. petiolare*	152,064	83,520	25,217	18,107	37.24	35.28	42.76	30.92	131	86	8	37
NC_034949	*C. quinoa*	152,099	83,582	25,204	18,106	37.24	35.29	42.74	30.96	131	86	8	37
NC_041201	*D. ambrosioides*	151,689	83,421	25,102	18,061	36.92	34.86	42.74	30.29	131	86	8	37
NC_042166	*D. botrys*	152,055	83,769	25,184	17,915	36.84	34.74	42.72	30.14	131	86	8	37
NC_041159	*D. pumilio*	151,962	83,758	25,230	17,741	36.95	34.84	42.74	30.44	131	86	8	37
NC_047226	*O. glauca*	151,655	83,579	25,080	17,913	36.87	34.73	42.77	30.35	131	86	8	37

* Represents the newly obtained chloroplast genome data in this study.

**Table 2:** Gene composition of the cp genome in *Chenopodium* and its related species.

Category	Group of Genes	Genes
Photosynthesis	Photosystem I	*psaA*, *psaB*, *psaC*, *psaI*, *psaJ*
Related genes	Photosystem II	*psbA*, *psbB*, *psbC*, *psbD*, *psbE*, *psbF*, *psbH*, *psbI*, *psbJ*, *psbK*,
		*psbL*, *psbM*, *psbN*, *psbT*, *psbZ*
	NADH oxidoreductase	*ndhA* *, *ndhB* *#, *ndhC*, *ndhD*, *ndhE*, *ndhF*, *ndhG*, *ndhH,*
		*ndhI*, *ndhJ*, *ndhK*
	Cytochrome b6/f complex	*petA*, *petB* *, *petD* *, *petG*, *petL*, *petN*
	ATP synthase	*atpA*, *atpB*, *atpE*, *atpF* *, *atpH*, *atpI*
	Rubisco	*rbcL*
	Assembly/stability	*ycf3* **, *ycf4*
	Cytochrome c synthesis	*ccsA*
Transcription and	Large subunit ribosomal proteins	*rpl14*, *rpl16* *, *rpl2* #, *rpl20*, *rpl22*, *rpl23* #, *rpl32*, *rpl33*, *rpl36*
Translation-related genes	Small subunit ribosomal proteins	*rps11*, *rps12* **#, *rps14*, *rps15*, *rps16* *, *rps18*, *rps19*, *rps2*, *rps3,*
		*rps4*, *rps7* #, *rps8*
	Transcription	*rpoA*, *rpoB*, *rpoC1* *, *rpoC2*
	Translation initiation factor	*infA*
	Ribosomal RNAs	*rrn16* #, *rrn23* #, *rrn4.5* #, *rrn5* #
	Transfer RNAs	*trnA-UGC* *#, *trnC-GCA*, *trnD-GUC*, *trnE-UUC*, *trnF-GAA,*
		*trnG-GCC*, *trnG-UCC* *, *trnH-GUG*, *trnI-CAU* #, *trnI-GAU* *#*,*
		*trnK-UUU* *, *trnL-CAA* #, *trnL-UAA* *, *trnL-UAG*, *trnM-CAU,*
		*trnN-GUU* #, *trnP-UGG*, *trnQ-UUG*, *trnR-ACG* #, *trnR-UCU,*
		*trnS-GCU*, *trnS-GGA*, *trnS-UGA*, *trnT-GGU*, *trnT-UGU*,
		*trnV-GAC* #, *trnV-UAC* *, *trnW-CCA*, *trnY-GUA*, *trnfM-CAU*
Other genes	RNA processing	*matK*
	Proteolysis	*clpP* **
	Carbon metabolism	*cemA*
	Fatty acid synthesis	*accD*
	Proteins with unknown functions	*ycf1* #, *ycf2* #

Gene *: Gene containing one intron; Gene **: Gene containing two introns; Gene #: Two gene copies in the IRs (*ycf1* located at the boundary of SSC and IRb is a pseudogene).

## Data Availability

All WGS data employed in this research were stored at the National Center for Biotechnology Information (NCBI), BioProject: PRJNA1027242, and the assembled cp genomes were stored under Genbank accession: OR374019-OR374024.

## Supplementary Materials

The following supporting information can be downloaded at: https://www.mdpi.com/article/10.3390/genes14122183/s1. Table S1: Comparison of the nucleotide diversity (Pi) of the cp genomes of the 16 *Chenopodium* and related species. Table S2: The GenBank information of the chloroplast genomes used to infer the *amaranthaceae* phylogeny. Table S3: Information on the primers used to identify the 10 closely related species of *Chenopodium*.
